# Explainable Artificial Intelligence Warning Model Using an Ensemble Approach for In-Hospital Cardiac Arrest Prediction: Retrospective Cohort Study

**DOI:** 10.2196/48244

**Published:** 2023-12-22

**Authors:** Yun Kwan Kim, Ja Hyung Koo, Sun Jung Lee, Hee Seok Song, Minji Lee

**Affiliations:** 1 Department of Research and Development Seers Technology Co, Ltd Pyeongtaek Republic of Korea; 2 Department of Brain and Cognitive Engineering Korea University Seoul Republic of Korea; 3 Department of Biomedical Software Engineering The Catholic University of Korea Gyeonggi Republic of Korea

**Keywords:** cardiac arrest prediction, ensemble learning, temporal pattern changes, cost-sensitive learning, electronic medical records

## Abstract

**Background:**

Cardiac arrest (CA) is the leading cause of death in critically ill patients. Clinical research has shown that early identification of CA reduces mortality. Algorithms capable of predicting CA with high sensitivity have been developed using multivariate time series data. However, these algorithms suffer from a high rate of false alarms, and their results are not clinically interpretable.

**Objective:**

We propose an ensemble approach using multiresolution statistical features and cosine similarity–based features for the timely prediction of CA. Furthermore, this approach provides clinically interpretable results that can be adopted by clinicians.

**Methods:**

Patients were retrospectively analyzed using data from the Medical Information Mart for Intensive Care-IV database and the eICU Collaborative Research Database. Based on the multivariate vital signs of a 24-hour time window for adults diagnosed with heart failure, we extracted multiresolution statistical and cosine similarity–based features. These features were used to construct and develop gradient boosting decision trees. Therefore, we adopted cost-sensitive learning as a solution. Then, 10-fold cross-validation was performed to check the consistency of the model performance, and the Shapley additive explanation algorithm was used to capture the overall interpretability of the proposed model. Next, external validation using the eICU Collaborative Research Database was performed to check the generalization ability.

**Results:**

The proposed method yielded an overall area under the receiver operating characteristic curve (AUROC) of 0.86 and area under the precision-recall curve (AUPRC) of 0.58. In terms of the timely prediction of CA, the proposed model achieved an AUROC above 0.80 for predicting CA events up to 6 hours in advance. The proposed method simultaneously improved precision and sensitivity to increase the AUPRC, which reduced the number of false alarms while maintaining high sensitivity. This result indicates that the predictive performance of the proposed model is superior to the performances of the models reported in previous studies. Next, we demonstrated the effect of feature importance on the clinical interpretability of the proposed method and inferred the effect between the non-CA and CA groups. Finally, external validation was performed using the eICU Collaborative Research Database, and an AUROC of 0.74 and AUPRC of 0.44 were obtained in a general intensive care unit population.

**Conclusions:**

The proposed framework can provide clinicians with more accurate CA prediction results and reduce false alarm rates through internal and external validation. In addition, clinically interpretable prediction results can facilitate clinician understanding. Furthermore, the similarity of vital sign changes can provide insights into temporal pattern changes in CA prediction in patients with heart failure–related diagnoses. Therefore, our system is sufficiently feasible for routine clinical use. In addition, regarding the proposed CA prediction system, a clinically mature application has been developed and verified in the future digital health field.

## Introduction

Critical illness was defined as the presence or potential development of organ dysfunction. Cardiac arrest (CA), a critical illness that affects patient safety, is the sudden cessation of cardiac function caused by specific abnormal events, such as ventricular arrhythmia, asystole, and pulseless electrical activity [1,2]. Previous studies have reported that at least one abnormal sign, such as respiratory distress or hemodynamic instability, occurs in 59.4% of patients within 1-4 hours before the onset of CA [3]. A previous study showed that early identification of the causes of CA improved patient survival by approximately 29% within the first hour of the episode and 19% at discharge [4]. Therefore, the early prediction of CA is important to allow for more time for clinical intervention, thereby reducing mortality.

Clinical decision support systems (CDSSs) are clinical computer systems that apply algorithms to patient information, use machine learning to evaluate clinical data, and provide clinical decision support [5,6]. These systems have been developed using electronic medical records to exploit various paradigms, such as the prediction of early cardiac events, heart failure (HF), and critical illness, for rapid response systems through real-time patient monitoring [7]. To improve the quality and speed of medical services, CA prediction and warning systems in intensive care units (ICUs) have been developed in the field of CDSSs [7]. These computer-based CA prediction algorithms provide new opportunities for clinicians to improve the accuracy of predicting CA events [8].

Several studies have used statistical methods for the early detection of CA [9-11]. Statistical methods generally use latent clinical features, including the simplified acute physiology score (SAPS)-II [9] and sequential organ failure assessment (SOFA) [12], which are calculated after the first day of ICU admission using data collected at a prespecified time frame. In addition, the modified early warning score (MEWS) [13] is a tool used by in-hospital care teams to identify early indicators of clinical deterioration and initiate early intervention and therapy.

Recently, machine learning approaches have been used to develop robust CA predictions for CDSSs. For example, Churpek et al [14] used a random forest (RF) classifier based on demographics, hospitalization histories, vital signs, and laboratory results extracted from a multicenter data set and obtained an area under the receiver operating characteristic curve (AUROC) of 0.83. Hong et al [15] used a clinical data set from a retrospective clinical study to apply an RF model. A clinical data set was collected from emergency department patients with CA at a tertiary academic hospital. They extracted the vital signs, sex, age, and primary concerns from the clinical data set. The proposed model achieved an AUROC of 0.97 and an area under the precision-recall curve (AUPRC) of 0.86. While their proposed model generally achieved more accurate CA prediction results than existing models, it relied excessively on features that were not commonly used during hospitalization and did not provide real-time predictions. Layeghian Javan et al [16] proposed a stacking method including RF, balanced bagging, and logistic regression (LR) to predict CA 1 hour in advance, and they obtained an AUROC of 0.82 using the Medical Information Mart for Intensive Care (MIMIC)-III [17]. Kwon et al [18] proposed a deep learning–based early warning system using a recurrent neural network (RNN) to assess risk scores using input vectors measured over 8 hours. They extracted vital signs from a retrospective multicenter cohort data set and obtained AUROC and AUPRC values of 0.85 and 0.04, respectively.

Statistical and machine learning techniques used in hospital settings for the early prediction of CA have certain limitations. First, current CA prediction algorithms for CDSSs suffer from low precision and high false alarm rates [19]. Second, a class imbalance problem exists in a skewed class distribution because CA events occur less frequently than in normal states [20]. Third, the influence of various characteristics on the results obtained from the model and decision support information must be determined [11]. An interpretable model that can provide this information has not yet been developed.

This study aimed to address these issues by proposing a framework for the early and accurate prediction of CA using CDSSs. We used an ensemble approach with gradient boosting ensemble of decision trees (LGB) classifiers to improve the overall precision of the CA prediction and reduce the false alarm rate. Furthermore, a cost-sensitive learning approach was considered to solve the imbalance problem regarding the class weights of CA events. In addition, the MIMIC-IV data set was used to show changes in feature importance according to changes in time for referencing clinical decisions [21].

## Methods

### Data Source

The MIMIC-IV database [21], which contains information on vital signs, laboratory tests, and procedural events of ICU patients, was used to develop and validate a CA prediction model using multivariate vital sign time series data of patients with HF. Specifically, this is a well-known single-center database that contains information on 46,520 patients admitted to the Beth Israel Deaconess Medical Center (BIDMC) between 2008 and 2019. Demographic data, International Classification of Diseases codes (IX), clinical modification codes, hourly vital signs and inputs or outputs, laboratory test and microbiological culture results, imaging data, treatment methods, medication administration, and survival statistics were included in the relevant records. In addition, MIMIC-IV [21] includes data from the clinical information system iMDsoft MetaVision. Compared to MIMIC-III, which extracts data from heterogeneous sources, this system provides more patient data and detailed information on procedure events, a main source of clinical information in ICUs [17]. Therefore, unlike MIMIC-III data, MIMIC-IV data [21] are homogeneous.

We used the eICU Collaborative Research Database (eICU-CRD) for external validation. The eICU-CRD is populated with data from more than 200,000 ICU admissions monitored across the United States by the eICU-CRD program developed by Philips Healthcare. The data in this collaborative database involve patients admitted to the ICU in 2014 and 2015.

### Ethical Considerations

The MIMIC-IV database and eICU-CRD are deidentified, transformed, and made available to researchers who have completed training in human research and signed a data use agreement. The Institutional Review Board at the BIDMC granted a waiver of informed consent and approved the MIMIC-IV database sharing initiative, and the eICU-CRD data were exempt from institutional review board approval with a waiver of informed consent [22,23].

### Problem Definition

The task in this study was to predict CA events 1 hour in advance. The input data contained the patient’s vital signs, the MEWS of temperature, and oxyhemoglobin saturation (SpO_2_) values from a 24-hour time window. The output is a binary vector, where each number represents the likelihood of a CA event in the next 1 hour. The primary outcomes comprised the AUROC and AUPRC scores, which were used to quantitatively check the prediction results for CA events 1 hour in advance. Next, we used the sensitivity, specificity, and F1-score as secondary outcomes to confirm any decrease in false alarms or missed CA events. In addition, we presented clinically interpretable decision support information.

### Prediction Model Framework

**Figure 1:**
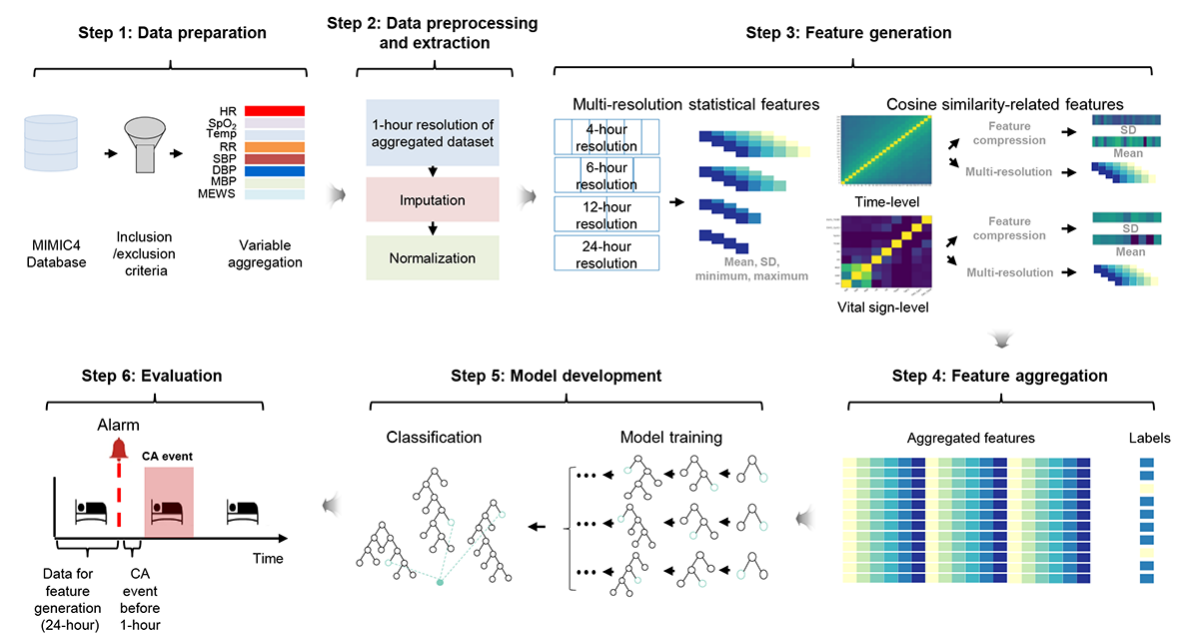
Overview of the proposed CA prediction framework. CA: cardiac arrest; DBP: diastolic blood pressure; HR: heart rate; MBP: mean blood pressure; MEWS: modified early warning score; MIMIC: Medical Information Mart for Intensive Care; RR: respiratory rate; SBP: systolic blood pressure; SpO_2_: oxyhemoglobin saturation; TEMP: temperature.

We suggest a framework for predicting CA 1 hour in advance. As shown in Figure 1, the proposed framework consists of 6 parts: data preparation, data preprocessing and extraction, feature generation, feature aggregation, model development, and evaluation. First, data were obtained from the MIMIC-IV database to construct a cohort that met the inclusion and exclusion criteria [21]. After filtering the inclusion and exclusion criteria, we extracted vital signs and calculated the MEWS through the vital signs. Then, in step 2, the features were processed and normalized after resampling the vital signs and MEWS at a resolution of 1 hour. Next, in step 3, 2 features were generated: statistical features and cosine similarity–based features. Multiresolution statistical features were generated using a sliding window–based statistical approach to segment each vital sign at 4, 6, 12, and 24 hours. The cosine similarity measure creates time-level and vital sign–level features that capture the degree of similarity in the changes in vital signs over time. Next, time-level and vital sign–level similarity matrices were used to calculate the mean and SD. In addition, a multiresolution statistical approach was used to extract time-level and vital sign–level similarity matrices to capture statistical similarity changes. In step 4, multiresolution statistical features, cosine similarity–based features, and labels were aggregated. Then, in step 5, we created an LGB classifier that is easy to implement and achieves good classification results in various medical tasks [24], containing different cost weights for each class. Finally, in step 6, the performance of the proposed model was measured using the evaluation metrics of precision, sensitivity, specificity, F1-score, AUROC, and AUPRC. Information about the open source and development code used is presented in Multimedia Appendix 1

#### Step 1: Data Preparation

**Figure 2:**
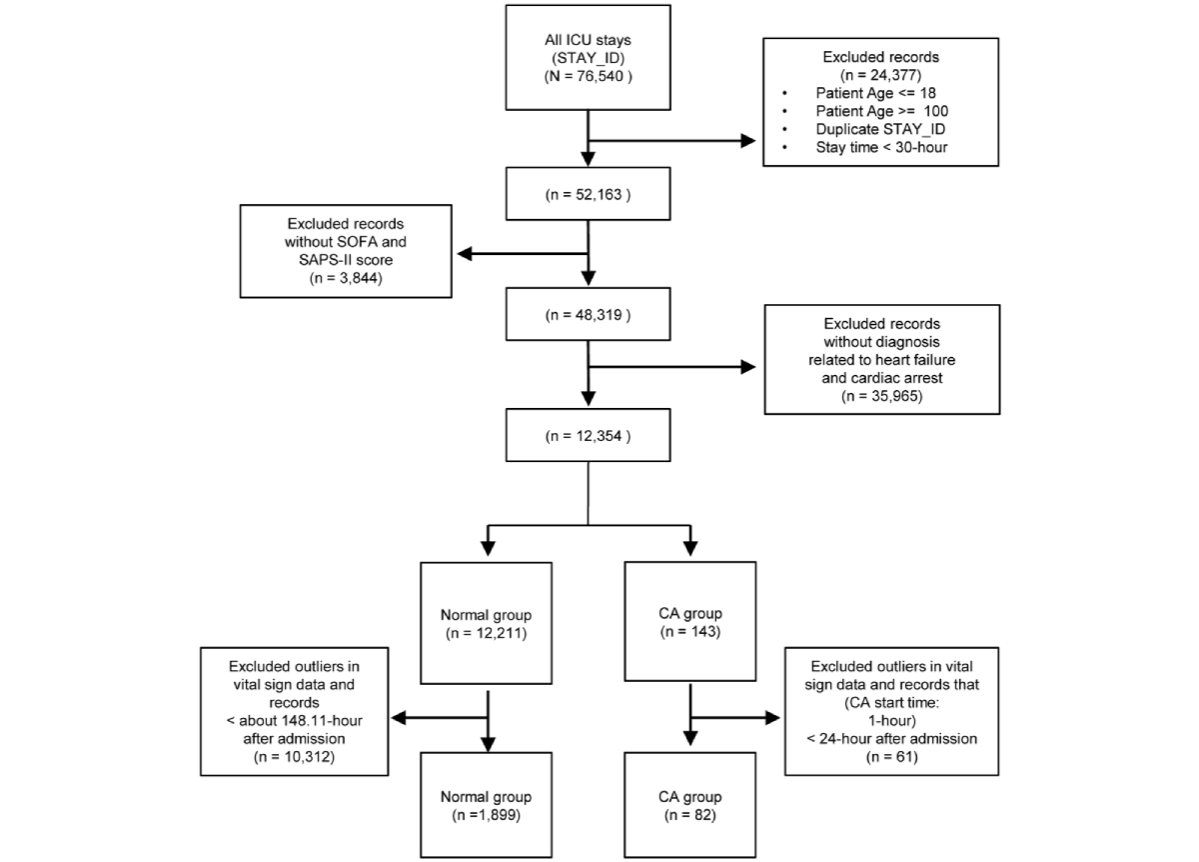
Patient inclusion and exclusion flow diagram for the Medical Information Mart for Intensive Care-IV database. CA: cardiac arrest; ICU: intensive care unit; n: number of stays; SAPS: simplified acute physiology score; SOFA: sequential organ failure assessment.

The inclusion and exclusion criteria were established to select the necessary data for CA prediction (Figure 2). After applying the inclusion and exclusion criteria, a cohort study was conducted. The study included patients aged >18 years and <100 years. HF is a major risk factor for sudden CA and a significant contributor to sudden CA mortality [25,26]. As CA occurs more frequently in patients with a history of HF or CA, we included the ICU stay of patients with these cardiovascular diseases in the cohort study. In the CA group, data on ICU stay were included if the vital sign data were not outliers and if any events that occurred 1 hour before CA occurred 24 hours after patient admission. In the normal group, data on ICU stay were included if the vital sign data were not outliers and if the admission time was longer than the average admission time in the CA group. Finally, an experimental database was created with 82 cases in the CA group and 1899 cases in the normal group.

#### Step 2: Data Preprocessing and Extraction

We collected data on the vital sign parameters, including heart rate (HR), systolic blood pressure (SBP), diastolic blood pressure (DBP), mean arterial pressure (MAP), temperature, respiratory rate (RR), and SpO_2_, of the patients from the experimental database. Vital sign parameters may be recorded with irregularly sampled time series data because of equipment malfunction and declining recipient response [27]. Prediction models are not designed to classify data with irregular samples from time series between groups. To solve this problem, the models require data collected at regular time intervals. We used the bucketing technique to solve the problem of irregularities in the time series [16]. We divided the 12- and 24-hour time windows into 12 and 24 sequential buckets of 1 hour each, respectively. The measured values within a bucket were averaged. As a result, each time series included 12 and 24 values at regular 1-hour intervals. When averaging within a bucket, if there was no time series value in the bucket, it was marked as null. To solve the problem of missing values as null, we used the last observation carried forward (LOCF) and backward (LOCB) imputation techniques [28]. The LOCF imputation technique is a technique in which previous nonmissing values are carried or copied forward and replaced with missing values. Similar to the LOCF method, the LOCB method replaces missing values by carrying or copying post-nonmissing values to the preceding missing values. Although we mainly used the LOCB method to impute missing values, the LOCF method was used when post values were missing, and then, missing values imputed previous nonmissing values. Additionally, we extracted the early warning score (EWS) for temperature and SpO_2_. We used the MEWS [13], which is a composite score commonly used by medical staff to determine illness severity. EWS observations were assigned a score between 0 and 3. The EWS calculated temperature and SpO_2_ every 1 hour. To remove outliers, the acceptable range of each variable was determined according to the opinions of medical experts. Values outside the acceptable range were eliminated. Then, we normalized each feature using z-score normalization because each column listing features has a different scale. We processed the database into an hourly time series with 12- and 24-hour time steps. Then, we combined the CA and normal (non-CA) groups to perform an imputation task.

#### Step 3: Feature Generation

##### Multiresolution Statistical Features

To capture the temporal history of the data, we created time windows of increasing size and extracted summary statistics across the multiresolution sliding window. Regarding the multiresolution sliding window–based statistical features, the input data were used to segment each vital sign at 4-, 6-, 12-, and 24-hour resolutions. All time-series segments of the vital sign data were aggregated as the mean, median, minimum, maximum, and SD of each feature.

##### Cosine Similarity–Based Features

We used a cosine similarity measure to capture the changes in the degree of similarity between vital signs over time. We then measured the degree of similarity in the changes in the vital sign features over time and the input data similarity degree of the changes in the vital sign types. To extract similarity features, we performed 3 steps. First, we extracted the cosine similarity matrix between the vital signs in the input time series data and their time steps in the vital signs. The similarity features at the time and vital sign levels were aggregated as the mean and SD in a single dimension. Additionally, multiresolution statistical features were extracted based on the similarity matrix at the time and vital sign levels to capture the statistical similarity changes in the mean and SD in that single dimension. Next, we created a weighted matrix multiplied by the raw vital sign matrix and cosine similarity–based features.

#### Step 4: Feature Aggregation

We aggregated multiresolution statistical features, cosine similarity–based features, and labels to derive better temporal features and inter-ICU generalizations from the model using vital signs and specific clinical latent scores. We then aggregated the variables into binary indicators, indicating the presence or absence of CA in a given class.

#### Step 5: Model Development

There are 3 approaches for handling the problem of class imbalance: data-, algorithm-, and hybrid-level approaches. We used an algorithm-level approach to address the extreme imbalances in our data set [29]. Specifically, we used cost-sensitive learning and the ensemble method of an LGB classifier to predict CA events within 1 hour of a patient’s ICU stay. The LGB classifier using cost-sensitive learning plays a role in reducing the bias or variance and improving the stability of machine learning algorithms [30,31]. Cost-sensitive learning was applied to penalize errors in the minority class of the CA group. Therefore, this method provides improved performance in applications where the medical data set has a highly skewed class distribution. Moreover, the LGB classifier uses cost-sensitive learning to reduce bias or variance and improve the stability of the machine learning algorithms. The minority classes were penalized at 100.

It is important to develop CA prediction models that increase sensitivity and reduce false-positive results in clinical settings. It is also important to develop an algorithm that uses a model with a sensitivity cutoff of 0.75 or higher for CA event prediction problems [32]. This is because it is important never to miss an event that is triggered, even if there is a false-positive result for the CA event. Precision and sensitivity are trade-offs, but these two metrics are important because they provide important information regarding the performance of the proposed method. Therefore, AUROC, a diagnostic index of models that considers both precision and sensitivity, is primarily used to compare the performance of prediction algorithms [33]. In summary, the model development phase focused on developing a model that maximizes sensitivity and AUROC.

After fitting the training set, the hyperparameter settings that maximized the AUROC in the validation set were used to generate predictions for the test set. If the AUROC of the validation set did not improve after 500 consecutive fitting iterations, the model was reset to its best iteration before premature termination. This model was then used for further analysis, as it was the best-performing model during system development. We set the number of trees to 1 to obtain the decision tree (DT) baseline, the weight of the CA class as a minority class to 100, and the learning rate to 0.04.

After tuning the hyperparameters, the Youden J statistic was used to select the optimal decision threshold value in the receiver operating characteristic curve of the proposed method [34]. We then calculated precision, sensitivity, and specificity using the decision threshold.

#### Step 6: Model Validation

We used the eICU-CRD to check the generalization ability of CA event predictions in more general settings. To conduct CA prediction events in a general ICU, we constructed the eICU-CRD data set, which had similar exclusion and inclusion criteria but was slightly different as follows. First, the target groups between the 2 databases were different. The MIMIC-IV database includes only cardiac-related diseases in the ICU, but the eICU-CRD covers all patients in the ICU. Second, the number of CA events per subject differed between the 2 databases. The CA event from the MIMIC-IV database is 1 per patient, while CA events from the eICU-CRD are multiple per patient. Because CA events may be multiple per patient in the clinical setting, we validated multiple events in the eICU-CRD (204 events/83 cases for the CA group). However, the rest of the inclusion and exclusion criteria were the same. Finally, we trained on the MIMIC-IV database with subjects with a higher risk of CA and validated with subjects in a more typical environment. In addition, we compared performance metrics between the proposed method and baseline models.

#### Step 7: Evaluation

##### K-Fold Cross-Validation

For internal validation, we used k-fold cross-validation, which avoids overfitting. In this study, k=10 was selected because it is a commonly used value [35]. Data are presented as mean±SD.

##### Baseline Models

We compared the CA event learning and prediction performance of the proposed model with those of 9 conventional ML methods: LR, k-nearest neighbors (KNN), DT, support vector machine (SVM), Gaussian naïve Bayes (GB), multilayer perceptron (MLP), RF, extreme gradient boosting ensemble of decision trees (XGB), and LGB. The details of the hyperparameters of the baseline models are listed in Multimedia Appendix 2.

##### Evaluation Metrics

We used the overall precision, sensitivity, specificity, AUROC, AUPRC, and Brier score values to evaluate model performance. The AUROC is a measure derived from sensitivity and specificity over different thresholds. For binary classification tasks, the AUROC ranges from 0.5 to 1, with values closer to 1 indicating better model performance. Clinical models are considered to have good or excellent discrimination ability if their AUROC is greater than 0.80 or 0.90. We also evaluated the sensitivity and specificity of each model using a series of validation runs. The AUPRC is useful for testing false alarm rates at different recalls and shows a relationship between precision (ie, 1 false alarm rate) and sensitivity [36]. The Brier score is the mean squared difference between the predicted probability and the actual outcome, with a lower Brier score indicating better calibration [37].

### Explainable Predictions

The Shapley additive explanation (SHAP) algorithm was applied to the proposed model to explain the features driving patient-specific predictions. The SHAP algorithm is an approach based on the game theory used to explain the performance of machine learning models, and it employs an additive feature attribution method to generate interpretable models [38,39]. SHAP is useful in explaining various supervised learning models and assigning importance values to each input variable for a specific prediction. This allowed us to interpret the decision-making process of the model and explain the prediction outcomes.

After extracting the impact of each feature using the proposed model, we summarized and visualized the 20 features with the highest mean values. In addition, the impact of the features over time was visualized as a heat map. Next, the features with the highest values were visualized according to changes over time.

### Statistical Analysis

Differences in patient characteristics, such as age, ICU length of stay, and vital signs, between the non-CA and CA groups were evaluated using independent *t*-tests. The performance metrics between the baseline and proposed models were tested using the Kruskal-Wallis test, and the Tukey honest significant difference test was used for the post-hoc analysis. In addition, the performance differences between the feature types, statistical features, cosine similarity–based features, and combined statistical and cosine similarity–based features were evaluated using the Kruskal-Wallis test for post-hoc analysis. A 5% significance level (*P*<.05) was used for all the analyses.

## Results

### Patient Characteristics

In the 24-hour time window, 1981 ICU stay cases (82 CA cases and 1899 non-CA cases) were included. The patient characteristics corresponding to these cases have been presented as means and SDs. The data are listed in Table 1. Independent sample *t*-tests were performed to analyze differences between the CA and non-CA groups. Age and ICU length of stay were not considered significant because their significance levels were greater than .05. Except for DBP, which was significant in both groups, the significance levels were less than .05. The details of patients in the ICU according to the inclusion of the 12-hour time window are listed in Multimedia Appendix 3. Furthermore, the eICU-CRD used for external validation included 9482 ICU stay cases (83 CA cases and 9399 non-CA cases) in the 24-hour time window. The details of patients in the ICU from the eICU-CRD according to the inclusion of the 24-hour time window are listed in Multimedia Appendix 4.

**Table 1:** Patient characteristics.

Characteristic			CA^a^ (n^b^=82)		Non-CA (n=1899)		*P* value
Age (years), mean (SD)			69.24 (13.60)		68.01 (13.71)		.43
ICU^c^ length of stay (h), mean (SD)			321.97 (336.65)		298.54 (286.20)		.54
**Vital signs, mean (SD)**							
	HR^d^	89.92 (16.41)		87.11 (17.22)		<.001	
	SpO_2_^e^	97.25 (3.85)		96.98 (3.11)		<.001	
	RR^f^	21.64 (5.50)		21.05 (5.79)		<.001	
	SBP^g^	111.98 (21.68)		117.81 (21.72)		<.001	
	DBP^h^	61.56 (13.86)		59.23 (14.28)		.17	
	MBP^i^	76.29 (14.62)		75.59 (14.88)		<.001	
	Temperature	37.08 (0.82)		36.93 (0.71)		<.001	

^a^CA: cardiac arrest.

^b^n: number of ICU stays.

^c^ICU: intensive care unit.

^d^HR: heart rate.

^e^SpO_2_: oxyhemoglobin saturation.

^f^RR: respiratory rate.

^g^SBP: systolic blood pressure.

^h^DBP: diastolic blood pressure.

^i^MBP: mean blood pressure.

### Evaluation of Model Performance

To investigate the effect of the proposed model in both the 24- and 12-hour time windows, we compared the performance of the model with that of the comparison models using 10-fold cross-validation of the binary class prediction results of the MIMIC-IV database. The same test database was used to ensure a fair comparison.

In the 24-hour time window obtained from the MIMIC-IV database, the proposed model achieved the best performance with mean AUROC and AUPRC values of 0.86±0.01 and 0.58±0.07, respectively, for predicting CA 1 hour in advance (Table 2; Multimedia Appendix 5). Next, we compared additional performance metrics in the 24-hour time window. The proposed model obtained the best performance with precision, sensitivity, specificity, and F1-score values of 0.68±0.04, 0.90±0.03, 0.90±0.04, and 0.72±0.04, respectively (Multimedia Appendix 5). The AUROC values indicated that the proposed model had statistically better performance than KNN, DT, SVM, GB, and RF when we conducted a comparison of the statistical analysis (*P*<.001; Multimedia Appendix 6). The proposed model outperformed the comparison models in the AUROC results.

We compared the precision, specificity, AUROC, and AUPRC based on a model with a sensitivity cutoff of 0.75 or higher because it is important for the CA prediction algorithm to avoid missing CA events [32]. Although the KNN and MLP classifiers obtained higher precision than the proposed method, the sensitivity of these classifiers was lower based on the sensitivity cutoff criteria. The proposed method achieved the highest precision among the compared models, including DT, RF, XGB, and LGB, with a sensitivity of 0.75 or higher. It also showed the highest specificity, AUROC, and AUPRC.

The proposed method outperformed the baseline models in terms of AUROC results in the 12-hour time window when we performed a comparison of the statistical AUROC results achieved by the baseline models and the proposed model in the 12-hour time window of the MIMIC-IV database (Multimedia Appendix 7).

**Table 2:** Results predicted by the proposed model for different time windows.

Window and feature		Precision, mean (SD)	Sensitivity, mean (SD)	Specificity, mean (SD)	F1-score, mean (SD)	AUROC^a^, mean (SD)	AUPRC^b^, mean (SD)
**12-hour window**							
	Statistical features	0.53 (0.00)	0.65 (0.02)	0.65 (0.02)	0.35 (0.01)	0.65 (0.02)	0.26 (0.05)
	Similarity features	0.54 (0.00)	0.71 (0.01)	0.71 (0.01)	0.41 (0.01)	0.71 (0.01)	0.38 (0.06)
	All statistical features	0.54 (0.00)	0.73 (0.02)	0.73 (0.02)	0.45 (0.01)	0.73 (0.02)	0.40 (0.05)
**24-hour window**							
	Statistical features	0.53 (0.00)	0.69 (0.03)	0.69 (0.03)	0.41 (0.01)	0.69 (0.03)	0.34 (0.09)
	Similarity features	0.57 (0.00)	0.84 (0.02)	0.84 (0.02)	0.56 (0.00)	0.84 (0.02)	0.52 (0.06)
	All statistical features	0.68 (0.04)	0.90 (0.03)	0.90 (0.04)	0.72 (0.04)	0.86 (0.02)	0.58 (0.07)

^a^AUROC: area under the receiver operating characteristic curve.

^b^AUPRC: area under the precision-recall curve.

### Effects of the Feature Set

The proposed framework comprises 3 types of feature sets: (1) statistical features, (2) cosine similarity–based features, and (3) a combination of statistical and cosine similarity–based features. In addition, various feature set experiments were conducted using the MIMIC-IV database to demonstrate the role and effectiveness of each feature set. We compared the performances of the feature set types using 10-fold cross-validation with the MIMIC-IV database to investigate the effect of each feature set on the proposed model.

We compared feature set types, including statistical features, cosine similarity–based features, and a combination of statistical and cosine similarity–based features, in 24- and 12-hour time windows. The performance metrics of most models using the 24-hour time window improved when the cosine similarity–based feature set was input into the model and the proposed method, with XGB and LGB obtaining statistically higher AUROC values (*P*<.001; Table 2; Multimedia Appendix 8). The performance metrics of most models using the 12-hour time window also improved when the cosine similarity–based feature set was input into the model and the proposed method (Table 2; Multimedia Appendix 9).

The combination of feature sets generated through the 24-hour time window improved the performance of the proposed model. Compared with its performance when employing the feature sets generated using the 24-hour time window, the proposed model achieved a lower performance when employing the feature sets generated using the 12-hour time window. Compared with its performance when using the statistical feature set, its performance was higher when using the cosine similarity–based features and a combination of feature sets. Therefore, we inferred that the cosine similarity–based feature set improved the performance of the proposed model when using the features generated by the 12- and 24-hour time windows.

### Effect of the Time Window

Different performance metrics were used to evaluate the prediction results of the proposed model with different feature sets, including statistical features, cosine similarity–based features, and a combination of statistical and cosine similarity–based features in both the 12- and 24-hour time windows, as shown in Table 2.

### External Validation of the Model

**Figure 3:**
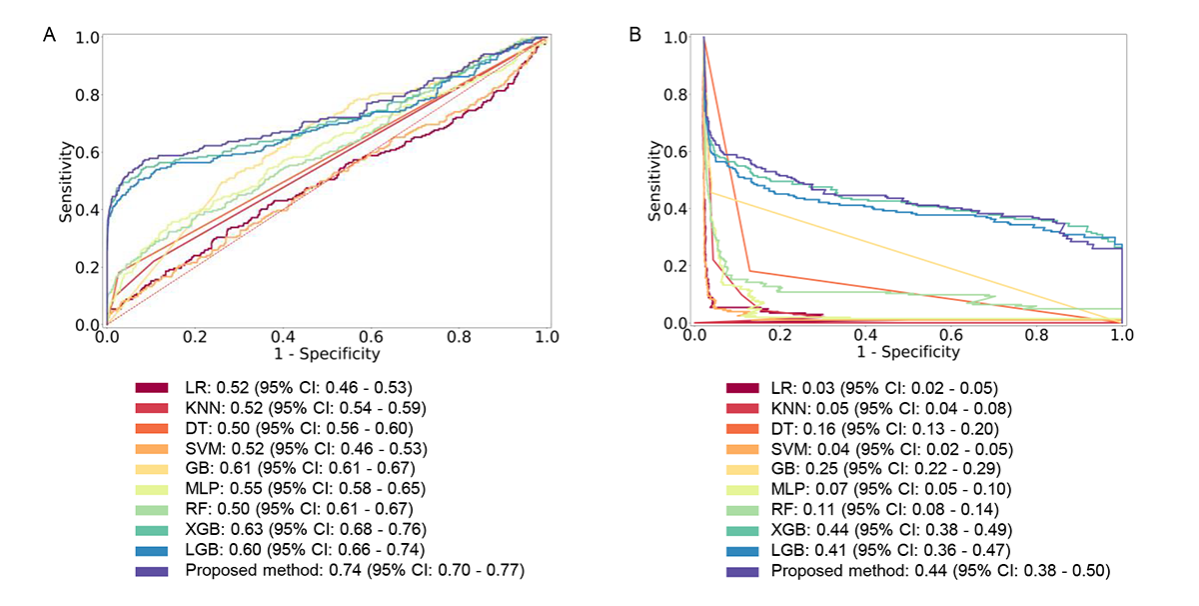
Comparison among baseline models and the proposed method using a 24-hour time window from the eICU Collaborative Research Database. (A) AUROC; (B) AUPRC. The baseline and proposed models were trained on the Medical Information Mart for Intensive Care-IV database. After the training procedure, we validated the baseline models and the proposed model to estimate generalization ability. We have presented 95% CIs after 1000 bootstrap iterations. AUPRC: area under the precision-recall curve; AUROC: area under the receiver operating characteristic curve; DT: decision tree; GB: Gaussian naïve Bayes; KNN: k-nearest neighbors; LGB: gradient boosting ensemble of decision trees; LR: logistic regression; MLP: multilayer perceptron; RF: random forest; SVM: support vector machine; XGB: extreme gradient boosting ensemble of decision trees.

We tested the proposed method and baseline models on the eICU-CRD as an independent database after training it on the MIMIC-IV database to measure its prediction of CA. We obtained an AUROC of 0.74 (95% CI 0.70-0.77) (Figure 3; Multimedia Appendix 10). The AUROCs of LR, KNN, DT, SVM, GB, MLP, RF, XGB, and LGB were 0.52 (95% CI 0.46-0.53), 0.52 (95% CI 0.54-0.59), 0.50 (95% CI 0.56-0.60), 0.52 (95% CI 0.46-0.53), 0.61 (95% CI 0.61-0.67), 0.55 (95% CI 0.58-0.65), 0.50 (95% CI 0.61-0.67), 0.63 (95% CI 0.68-0.76), and 0.60 (95% CI 0.66-0.74), respectively (Figure 3; Multimedia Appendix 10). Therefore, the AUROC results using the proposed method were higher than the AUROC results using comparison methods. In addition, the results of precision, sensitivity, F1-score, specificity, Brier score, and AUPRC were higher using the proposed method than using the comparison methods (Multimedia Appendix 10). This indicated that the proposed method outperformed comparison models on the eICU-CRD and successfully pulled features from external validation.

### Clinical Interpretability

**Figure 4:**
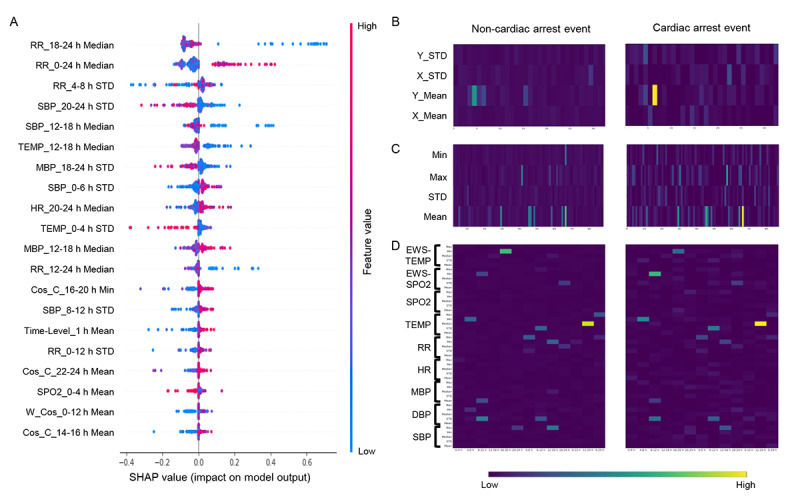
Clinical interpretability results. (A) Global feature impact values produced by the proposed model. (B) Cosine similarity feature set between the non-CA and CA groups. (C) Multiresolution statistical features based on the cosine similarity matrix between the non-CA and CA groups. (D) Statistical feature set between the non-CA and CA groups. C: channel-level average; CA: cardiac arrest; Cos: cosine similarity; DBP: diastolic blood pressure; HR: heart rate; MBP: mean blood pressure; MEWS: modified early warning score; MIMIC: Medical Information Mart for Intensive Care; RR: respiratory rate; SBP: systolic blood pressure; SHAP: Shapley additive explanation; SpO2: oxyhemoglobin saturation; TEMP: temperature; W: weighted matrix.

We used SHAP [38] values to evaluate the influence of each feature on the proposed model output. Positive and negative SHAP values indicated an increase and decrease in the prediction score, respectively. Figure 4 shows the top 20 features of the proposed model based on the SHAP values.

Regarding the impact of the model as a global aspect, RR_18-24 h_Median and RR_0-24 h_Median had relatively significant impacts on the performance of the proposed method. In addition, the 20 most influential features were created using the time-step data obtained after 12 hours.

Regarding cosine similarity features, the mean values of HR, RR, and SpO_2_ in the proposed model differed between the non-CA and CA groups. The effect of the time-level cosine similarity features changed between the non-CA and CA groups after 12 hours. Furthermore, the cosine similarity features for vital sign levels resulted in changes in HR, RR, and SpO_2_ values between the non-CA and CA groups.

Regarding the multiresolution statistical features based on the sliding window, temperature, EWS-temperature, SpO_2_, and EWS-SpO_2_ had different effects on the proposed model. Both the minimum value of EWS-temperature in the 16 to 24-hour window and the minimum value of EWS-SpO_2_ in the 8 to 12-hour window differed between the non-CA and CA groups.

### Comparison With Existing Research

Table 3 lists a comprehensive performance comparison between the CA prediction results of the proposed method and those of existing models. Churpek et al [14] used a clinical database to identify CA events at a given time point using an RF classifier and obtained an AUROC of 0.83. The time since ward admission, demographics, hospitalization history, vital signs, and laboratory test results were considered. Kwon et al [18] proposed an n-RNN for predicting CA events using vital sign information. Their model achieved AUROC and AUPRC values of 0.85 and 0.04, respectively. Layeghian Javan et al [16] suggested that a stacking ensemble model could predict CA 1 hour in advance. Their model used time intervals and statistical features generated by vital signs and latent clinical data from MIMIC-III and achieved an AUROC of 0.82.

The proposed method uses data from the MIMIC-IV database to generate statistical and cosine similarity–based feature sets. Using a combination of statistical and cosine similarity–based feature sets, the proposed method achieved AUROC and AUPRC values of 0.86 and 0.58, respectively. As listed in Table 3, the proposed model outperformed the existing models.

Considering the latest studies, Layeghian Javan et al [16] reported a precision of 0.19, sensitivity of 0.77, and AUROC of 0.82. In addition, Kwon et al [18] showed a precision of 0.05, sensitivity of 0.75, AUROC of 0.85, and AUPRC of 0.04 (Multimedia Appendix 11). In summary, our method had a higher precision (0.49), higher sensitivity (0.13), and higher AUROC (0.04) compared with the method of Layeghian Javan et al [16]. The proposed method showed high performance in terms of precision, sensitivity, AUROC, and AUPRC compared with the method of Kwon et al [18]. Therefore, the proposed model exhibited higher precision, sensitivity, specificity, F1-score, AUROC, and AUPRC than those of recent studies.

**Figure 5:**
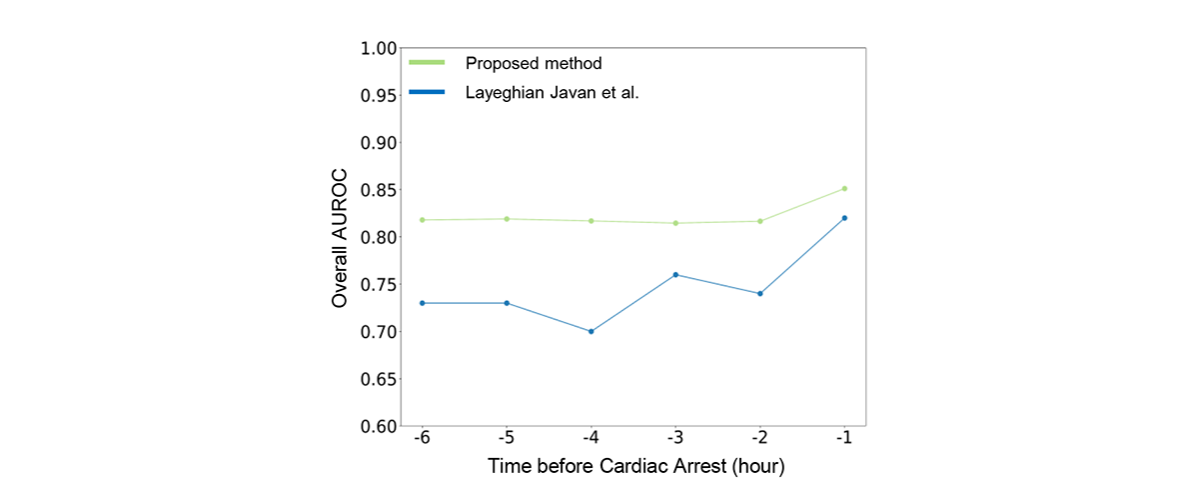
Comparison of AUROC values achieved by the proposed model and a state-of-the-art model. The light green line indicates the proposed model, while the blue line represents the method proposed by Layeghian Javan et al [16]. AUROC: area under the receiver operating characteristic curve.

In a comparison of the AUROC values for CA prediction up to 6 hours in advance, the proposed model achieved an AUROC over 0.80, whereas the model of Layeghian Javan et al [16] achieved an AUROC under 0.80. In addition, the proposed model obtained a higher AUROC for the prediction of CA 1 hour in advance (Figure 5).

**Table 3:** Results comparing the prediction performance between the proposed model and state-of-the-art models.

Author	Year	Group	Database	Features	Classifier	Explainable	Before CA^a^	Performance
Churpek et al [14]	2016	Non-CA: 253,547; CA: 424	Clinical database	Time since ward admission, demographics, hospitalization history, vital signs, and laboratory results	RF^b^	Yes	0 h, current point	AUROC^c^=0.83
Kwon et al [18]	2018	Non-CA: 45,539; CA: 396	Clinical database	Vital signs	RNN^d^	No	0 h, current point	AUROC=0.85; AUPRC^e^=0.04
Layeghian Javan et al [16]	2019	Non-CA: 2681; CA: 79	MIMIC^f^-III [17]	Time interval and statistical features using vital signs and clinical latent features	Stacking	No	1 h	AUROC=0.82
Proposed method	N/A^g^	Non-CA: 1899; CA: 82	MIMIC-IV [21]	Cosine similarity and statistical features using vital signs and clinical latent features	LGB^h^	Yes	1 h	AUROC=0.86 AUPRC=0.58

^a^CA: cardiac arrest.

^b^RF: random forest.

^c^AUROC: area under the receiver operating characteristic curve.

^d^RNN: recurrent neural network.

^e^AUPRC: area under the precision-recall curve.

^f^MIMIC: Medical Information Mart for Intensive Care.

^g^N/A: not applicable.

^h^LGB: gradient boosting ensemble of decision trees.

## Discussion

### Principal Findings

Clinicians can use the proposed model to make clinical decisions for patients with HF-related diagnoses in the ICU, providing rapid response services more accurately than those in previous studies. In this study, we developed and validated an ensemble approach–based model capable of predicting CA events 1 hour in advance. The prediction performance of the proposed model was considerably better than that of conventional machine learning models used for patients requiring ICU support. Therefore, the number of in-hospital CA events and deaths could be reduced. In addition, the proposed method obtained better prediction performance up to 6 hours in advance, allowing clinicians to be better prepared for in-hospital CA events.

Kwon et al [18] solved a real-time challenge using deep learning models and achieved high AUROC scores. However, owing to the black-box nature of these models, the relationship between the prediction results and features cannot be understood, making them undesirable for clinical decision support. Layeghian Javan et al [16] used an ensemble method with the stacking method to achieve a high AUROC value; however, their method could not provide a relationship between the prediction results and features.

Regarding CA prediction using existing machine learning methods, cosine similarity–based features led to statistically higher performance in terms of AUROC, AUPRC, and specificity than statistical features (all *P*<.001). The best performance was observed when the proposed LGB model used cosine similarity–based features. Moreover, when the other models and this feature set were combined, a statistically better performance was obtained than when only the statistical feature set was used (all *P*<.001). Therefore, cosine similarity–based features can play an important role in predicting the occurrence of CA.

Several noteworthy insights were obtained regarding the clinical interpretability results of the proposed model. First, we observed that the time-level cosine similarity–based features changed after 12 hours in the CA group but not in the non-CA group (Figure 4B and C). This result is consistent with the difference in the significance of the changes in temporal silence features between the non-CA and CA groups at 12 hours [3]. Specifically, the instability of at least one vital sign 1-4 hours before CA was consistent with the difference in the significance of changes in temporal silence features between the non-CA and CA groups at 12 hours [3] (Figure 4D). Second, vital sign–level features, including HR, RR, and SpO_2_, differed in their level of correlation with other vital sign data in the 24-hour time window (Figure 4B). This is consistent with the finding that changes in the temporal pattern of vital signs become irregular before CA occurs [3,40]. These results were consistent with the neuroscientific results. After being fed into the proposed model, the similarity change and correlation information of vital sign data over time showed a statistically significant improvement in performance based on the relationship between the statistical features and predictive power. This indicates that the proposed model is more useful for providing accurate CA predictions in a target population. Additionally, for patients with an HF-related diagnosis, the information extracted using a statistical method for temporal patterns from each vital sign was not significant in the CA prediction results. However, the information extracted using the cosine similarity–based feature set was significant, indicating that it provided valuable information for predicting CA.

### Strengths

This study has several strengths. First, we adopted widely applied machine learning models and model evaluation techniques that have rarely been applied to evaluate the clinical predictive ability of these machine learning models. Second, we tuned the hyperparameter values for each machine learning model identified through an iterative grid search. It was verified that hyperparameter tuning can improve the performance of these models. We proposed an interpretable and calibrated ensemble approach using LGB with different cost weights for each class to predict CA events within 1 hour. Compared with baseline models that are widely used in related clinical applications, the proposed model achieved the highest AUROC values and provided a statistically higher performance (all *P*<.001). Our proposed method achieved an AUROC exceeding 0.8 for predicting CA 6 hours in advance. Therefore, clinicians have sufficient time to respond to CA events when using the proposed model. Cosine similarity–based features statistically improved the performance of all models (all *P*<.001). The results revealed that the cosine similarity–based features of vital signs and EWSs greatly supported the prediction of CA events in patients with HF.

Our proposed CA prediction system significantly reduced false alarm rates and showed high performance in terms of precision, AUROC, and AUPRC compared with comparative models for validated internal and external data sets, and thus, it can be applied to ICU patients. In addition, our framework can explain which features among the vital signs input into the CA prediction system generate alerts to medical staff through feature importance analysis. This can easily help medical staff judge and improve the reliability of machine learning results. Therefore, our CA prediction system is considered to have reached clinical maturity and is being used and verified for everyday clinical use.

### Limitations

This study had several limitations. An ensemble approach based on gradient boosting was developed without feature screening. However, as indicated by the high-performance results, this approach did not significantly affect the model’s performance. Even though the proposed method showed higher precision, sensitivity, specificity, F1-score, AUROC, and AUPRC for external validation, the precision was still low, which is considered a limitation of our study. Further work is needed to propose feature generation methods and models that can further improve precision while maintaining AUROC and sensitivity. In the future, the proposed model could be optimized for feature screening. Nevertheless, as mentioned in the discussion section, the prediction model of this study has good potential for clinical applicability in CDSSs and early interventions. Accessibility and user ability can be improved using a user-centered CDSS or web-based application based on the proposed model.

### Conclusions

In this study, we evaluated the performance of an explainable artificial intelligence warning model using an ensemble technique in an ICU population. The proposed model incorporated statistical and cosine similarity–based features from vital signs in the 24-hour time window and achieved a high AUROC value for early CA diagnosis. The proposed model attempted to predict CA events every 1 hour. The SHAP value was used to explain overall and time-to-time relevance. These clinical interpretability results can aid doctors in making clinical decisions by providing insights into the links between predictive findings and characteristics. These findings indicate that the proposed technique outperformed other comparable models in terms of CA prediction in ICU settings.

## Appendix

Multimedia Appendix 1 Source code information.

Multimedia Appendix 2 Details about the hyperparameters of the baseline models.

Multimedia Appendix 3 Additional details on patients in the intensive care unit according to the inclusion of the 12-hour time window.

Multimedia Appendix 4 Additional details on patients from the eICU Collaborative Research Database according to the inclusion of the 24-hour time window.

Multimedia Appendix 5 Comparison results of additional performance metrics between the proposed method and baseline models in the 24-hour time window from the Medical Information Mart for Intensive Care-IV database.

Multimedia Appendix 6 Statistical comparison results of area under the receiver operating characteristic curve between the proposed method and the other classifiers using the 24-hour time window from the Medical Information Mart for Intensive Care-IV database.

Multimedia Appendix 7 Statistical comparison results of the area under the receiver operating characteristic curve between the proposed method and the other classifiers using the 12-hour time window from the Medical Information Mart for Intensive Care-IV database.

Multimedia Appendix 8 Performance metrics of most models using the 24-hour time window.

Multimedia Appendix 9 Performance metrics of most models using the 12-hour time window.

Multimedia Appendix 10 Comparison results of performance metrics between the proposed method and the baseline models using the eICU Collaborative Research Database.

Multimedia Appendix 11 Comparison results of performance metrics between the proposed method and methods in recent studies.

## Abbreviations

AUPRC: area under the precision-recall curve
AUROC: area under the receiver operating characteristic curve
CA: cardiac arrest
CDSS: clinical decision support system
DBP: diastolic blood pressure
DT: decision tree
eICU-CRD: eICU Collaborative Research Database
EWS: early warning score
GB: Gaussian naïve Bayes
HF: heart failure
HR: heart rate
ICU: intensive care unit
KNN: k-nearest neighbors
LGB: gradient boosting ensemble of decision trees
LOCB: last observation carried backward
LOCF: last observation carried forward
LR: logistic regression
MEWS: modified early warning score
MIMIC: Medical Information Mart for Intensive Care
MLP: multilayer perceptron
RF: random forest
RNN: recurrent neural network
RR: respiratory rate
SAPS: simplified acute physiology score
SBP: systolic blood pressure
SHAP: Shapley additive explanation
SOFA: sequential organ failure assessment
SpO2: oxyhemoglobin saturation
SVM: support vector machine
XGB: extreme gradient boosting ensemble of decision trees

## Data Availability

The Medical Information Mart for Intensive Care-IV database is available online upon request at the repository PhysioNet [41]. The eICU Collaborative Research Database is freely available through PhysioNet [42].
