# Supplementary material for: Explainable Artificial Intelligence Warning Model Using an Ensemble Approach for In-Hospital Cardiac Arrest Prediction: Retrospective Cohort Study
Source: J Med Internet Res. 2023 Dec 22;25:e48244. doi: 10.2196/48244 (PMC10770782; doi:10.2196/48244)
Supplement: Multimedia Appendix 1 [file jmir_v25i1e48244_app1.docx]

**Multimedia Appendix 1.** Source code information.

Source code generated and used for this study is publicly available for download at https://github.com/yunkwankim/CAP. Source code for scikit-learn packages of python is freely available at https://scikit-learn.org/stable/. Source code for SHAP is from https://shap.readthedocs.io/en/latest/.
