# Supplementary material for: Explainable Artificial Intelligence Warning Model Using an Ensemble Approach for In-Hospital Cardiac Arrest Prediction: Retrospective Cohort Study
Source: J Med Internet Res. 2023 Dec 22;25:e48244. doi: 10.2196/48244 (PMC10770782; doi:10.2196/48244)
Supplement: Multimedia Appendix 2 [file jmir_v25i1e48244_app2.docx]

**Multimedia Appendix 2.** Details about the hyperparameters of the baseline models.

| **Model** | **Hyperparameter** | **Class Weight**  **(Cardiac Arrest Group)** |
| --- | --- | --- |
| **LR**^a^ | L2 regularization  C = 1 | 150 |
| **KNN**^b^ | K = 5 | - |
| **DT**^c^ | Criterion = Gini  Maximum depth = 1 | 150 |
| **SVM**^d^ | Kernel = Linear kernel  C = 1 | 150 |
| **GB**^e^ | - | - |
| **MLP**^f^ | Optimizer = Adam  Hidden Layer = 100  Learning Rate = 0.001 | - |
| **RF**^g^ | Number of decision trees = 200 | 150 |
| **XGB**^h^ | Extreme profundity of trees = 1  Quantity of helping stages = 450  Learning rate = 0.04 | 150 |
| **LGB**^i^ | Extreme profundity of trees = 1  Quantity of helping stages = 550  Learning rate = 0.04 | 150 |

^a^LR: logistic regression

^b^KNN: k-nearest neighbors

^c^DT: decision tree

^d^SVM: support vector machine

^e^GB: Gaussian naïve Bayes

^f^MLP: multilayer perceptron

^g^RF: random forest

^h^XGB: extreme gradient boosting ensemble of decision trees

^i^LGB: gradient boosting ensemble of decision trees
