# Supplementary material for: Explainable Artificial Intelligence Warning Model Using an Ensemble Approach for In-Hospital Cardiac Arrest Prediction: Retrospective Cohort Study
Source: J Med Internet Res. 2023 Dec 22;25:e48244. doi: 10.2196/48244 (PMC10770782; doi:10.2196/48244)
Supplement: Multimedia Appendix 3 [file jmir_v25i1e48244_app3.docx]

**Multimedia Appendix 3.** Additional details on patients in the intensive care unit according to the inclusion of the 12-hour time window.

|  | **CA**^a^ **(n**^i^ **=92)** | **Non-CA (n=1899)** | ***P* value** |
| --- | --- | --- | --- |
| **Age (years), mean (SD)** | 69.29 (13.52) | 68.01 (13.71) | .38 |
| **ICU**^b^ **Length of Stay (hour)** | 295.56  (327.85) | 298.54  (286.20) | .93 |
| **Vital Signs** |  |  |  |
| **HR**^c^ | 89.94 (16.39) | 87.11 (17.22) | <.001 |
| **SpO_2_**^d^ | 97.24 (3.86) | 96.98 (3.11) | < .001 |
| **RR**^e^ | 21.63 (5.50) | 21.05 (5.79) | < .001 |
| **SBP**^f^ | 111.79 (21.67) | 117.81 (21.72) | < .001 |
| **DBP**^g^ | 61.46 (13.86) | 59.23 (14.28) | < .001 |
| **MBP**^h^ | 76.17 (14.63) | 75.59 (14.88) | < .001 |
| **Temperature** | 37.09 (0.83) | 36.93 (0.71) | < .001 |

The patient characteristics were presented as means and standard deviations. Independent-sample t-tests were performed to analyze differences between the cardiac arrest (CA) and non-CA patient groups.

^a^CA: cardiac arrest

^b^ICU: intensive care unit

^c^HR: heart rate

^d^SpO_2_: oxyhemoglobin saturation

^e^RR: respiratory rate

^f^SBP: systolic blood pressure

^g^DBP: diastolic blood pressure

^h^MBP: mean blood pressure

^i^n: number of ICU stays
