# Supplementary material for: Explainable Artificial Intelligence Warning Model Using an Ensemble Approach for In-Hospital Cardiac Arrest Prediction: Retrospective Cohort Study
Source: J Med Internet Res. 2023 Dec 22;25:e48244. doi: 10.2196/48244 (PMC10770782; doi:10.2196/48244)
Supplement: Multimedia Appendix 4 [file jmir_v25i1e48244_app4.docx]

**Multimedia Appendix 4.** Additional details on patients from the eICU Collaborative Research Database according to the inclusion of the 24-hour time window.

|  | **CA**^a^ **(n**^i^ **=83)** | **Non-CA (n=9399)** | ***P* value** |
| --- | --- | --- | --- |
| **Age (years), mean (SD)** | 60.06 (15.48) | 62.40 (17.12) | .17 |
| **ICU**^b^ **Length of Stay (hour)** | 200.19  (185.21) | 124.46  (143.87) | < .001 |
| **Vital Signs** |  |  |  |
| **HR**^c^ | 88.23 (19.01) | 86.59 (17.60) | < .001 |
| **SpO_2_**^d^ | 96.78 (4.14) | 96.41 (3.70) | < .001 |
| **RR**^e^ | 20.41 (5.56) | 19.73 (5.05) | < .001 |
| **SBP**^f^ | 119.06 (21.82) | 124.67 (21.92) | < .001 |
| **DBP**^g^ | 64.10 (13.79) | 68.01 (14.26) | < .001 |
| **MBP**^h^ | 79.45 (15.39) | 83.91 (15.87) | < .001 |
| **Temperature** | 36.94 (0.90) | 36.87 (0.56) | < .001 |

The patient characteristics were presented as means and standard deviations. Independent-sample t-tests were performed to analyze differences between the CA and non-CA patient groups. Age was not considered significant with a significance level greater than 0.05. Length of ICU stay and vital signs were significant in both groups, with significance levels less than 0.05.

^a^CA: cardiac arrest

^b^ICU: intensive care unit

^c^HR: heart rate

^d^SpO_2_: oxyhemoglobin saturation

^e^RR: respiratory rate

^f^SBP: systolic blood pressure

^g^DBP: diastolic blood pressure

^h^MBP: mean blood pressure

^i^n: number of ICU stays
