# Supplementary material for: Explainable Artificial Intelligence Warning Model Using an Ensemble Approach for In-Hospital Cardiac Arrest Prediction: Retrospective Cohort Study
Source: J Med Internet Res. 2023 Dec 22;25:e48244. doi: 10.2196/48244 (PMC10770782; doi:10.2196/48244)
Supplement: Multimedia Appendix 5 [file jmir_v25i1e48244_app5.docx]

**Multimedia Appendix 5.** Comparison results of additional performance metrics between the proposed method and baseline models in the 24-hour time window from the Medical Information Mart for Intensive Care-IV database.

|  | **Precision (SD)** | **Sensitivity (SD)** | **F1-score (SD)** | **Specificity (SD)** | **AUROC (SD)** | **AUPRC (SD)** |
| --- | --- | --- | --- | --- | --- | --- |
| **LR**^a^ | 0.61  (0.02) | 0.77  (0.05) | 0.65  (0.03) | 0.77  (0.01) | 0.77  (0.05) | 0.34  (0.07) |
| **KNN**^b^ | 0.75  (0.06) | 0.58  (0.02) | 0.61  (0.03) | 0.58  (0.00) | 0.58  (0.02) | 0.35  (0.06) |
| **DT**^c^ | 0.02  (0.00) | 0.50  (0.00) | 0.04  (0.00) | 0.50  (0.00) | 0.50  (0.00) | 0.41  (0.03) |
| **SVM**^d^ | 0.75  (0.07) | 0.76  (0.07) | **0.75**  (0.07) | 0.76  (0.01) | 0.76  (0.07) | 0.50  (0.14) |
| **GB**^e^ | 0.54  (0.00) | 0.68  (0.03) | 0.52  (0.01) | 0.68  (0.01) | 0.68  (0.03) | 0.31  (0.03) |
| **MLP**^f^ | **0.80**  (0.07) | 0.74  (0.07) | 0.75  (0.07) | 0.74  (0.00) | 0.74  (0.07) | 0.55  (0.12) |
| **RF**^g^ | 0.52  (0.00) | 0.59  (0.01) | 0.21  (0.01) | 0.59  (0.02) | 0.59  (0.01) | 0.43  (0.06) |
| **XGB**^h^ | 0.55  (0.00) | 0.79  (0.01) | 0.49  (0.00) | 0.79  (0.01) | 0.79  (0.01) | 0.53  (0.06) |
| **LGB**^i^ | 0.56  (0.00) | 0.83  (0.02) | 0.54  (0.01) | 0.83  (0.01) | 0.83  (0.02) | 0.55  (0.07) |
| **Proposed method** | 0.68  (0.04) | **0.90**  (0.03) | 0.72  (0.04) | **0.90**  (0.04) | **0.86**  (0.01) | **0.58**  (0.07) |

^a^LR: logistic regression

^b^KNN: k-nearest neighbors

^c^DT: decision tree

^d^SVM: support vector machine

^e^GB: Gaussian naïve Bayes

^f^MLP: multilayer perceptron

^g^RF: random forest

^h^XGB: extreme gradient boosting ensemble of decision trees

^i^LGB: gradient boosting ensemble of decision trees
