# Supplementary material for: Explainable Artificial Intelligence Warning Model Using an Ensemble Approach for In-Hospital Cardiac Arrest Prediction: Retrospective Cohort Study
Source: J Med Internet Res. 2023 Dec 22;25:e48244. doi: 10.2196/48244 (PMC10770782; doi:10.2196/48244)
Supplement: Multimedia Appendix 6 [file jmir_v25i1e48244_app6.docx]

**Multimedia Appendix 6.** Statistical comparison results of area under the receiver operating characteristic curve between the proposed method and the other classifiers using the 24-hour time window from the Medical Information Mart for Intensive Care-IV database.

| **Classifier** | **95% CI^k^** | | ***P* value** |
| --- | --- | --- | --- |
|  | **Lower limit** | **Upper limit** |  |
| **Proposed method vs LR**^b^ | -0.09 | 0.25 | .85 |
| **Proposed method vs KNN**^c^ | 0.11 | 0.45 | <.001 |
| **Proposed method vs DT**^d^ | 0.18 | 0.53 | <.001 |
| **Proposed method vs SVM**^e^ | -0.08 | 0.27 | .71 |
| **Proposed method vs GB**^f^ | 0.01 | 0.35 | .03 |
| **Proposed method vs MLP**^g^ | -0.06 | 0.29 | .49 |
| **Proposed method vs RF**^h^ | 0.09 | 0.44 | <.001 |
| **Proposed method vs XGB**^i^ | -0.17 | 0.18 | .90 |
| **Proposed method vs LGB**^j^ | -0.10 | 0.24 | .90 |

^a^MIMIC: medical information mart for intensive care

^b^LR: logistic regression

^c^KNN: k-nearest neighbors

^d^DT: decision tree

^e^SVM: support vector machine

^f^GB: Gaussian naïve Bayes

^g^MLP: multilayer perceptron

^h^RF: random forest

^i^XGB: extreme gradient boosting ensemble of decision trees

^j^LGB: gradient boosting ensemble of decision trees

^k^CI: confidence interval
