# Supplementary material for: Explainable Artificial Intelligence Warning Model Using an Ensemble Approach for In-Hospital Cardiac Arrest Prediction: Retrospective Cohort Study
Source: J Med Internet Res. 2023 Dec 22;25:e48244. doi: 10.2196/48244 (PMC10770782; doi:10.2196/48244)
Supplement: Multimedia Appendix 7 [file jmir_v25i1e48244_app7.docx]

**Multimedia Appendix 7.** Statistical comparison results of the area under the receiver operating characteristic curve between the proposed method and the other classifiers using the 12-hour time window from the Medical Information Mart for Intensive Care-IV database.

| **Classifier** | **95% CI^k^** | | ***P* value** |
| --- | --- | --- | --- |
|  | **Lower limit** | **Upper limit** |  |
| **Proposed method vs LR**^b^ | -0.18 | 0.14 | .90 |
| **Proposed method vs KNN**^c^ | 0.01 | 0.32 | .03 |
| **Proposed method vs DT**^d^ | 0.06 | 0.38 | <.001 |
| **Proposed method vs SVM**^e^ | -0.21 | 0.11 | .90 |
| **Proposed method vs GB**^f^ | -0.12 | 0.20 | .90 |
| **Proposed method vs MLP**^g^ | -0.19 | 0.12 | .90 |
| **Proposed method vs RF**^h^ | 0.04 | 0.36 | <.001 |
| **Proposed method vs XGB**^i^ | -0.17 | 0.14 | .90 |
| **Proposed method vs LGB**^j^ | -0.13 | 0.18 | .90 |

^a^MIMIC: medical information mart for intensive care

^b^LR: logistic regression

^c^KNN: k-nearest neighbors

^d^DT: decision tree

^e^SVM: support vector machine

^f^GB: Gaussian naïve Bayes

^g^MLP: multilayer perceptron

^h^RF: random forest

^i^XGB: extreme gradient boosting ensemble of decision trees

^j^LGB: gradient boosting ensemble of decision trees

^k^CI: Confidence interval
