# Supplementary material for: Explainable Artificial Intelligence Warning Model Using an Ensemble Approach for In-Hospital Cardiac Arrest Prediction: Retrospective Cohort Study
Source: J Med Internet Res. 2023 Dec 22;25:e48244. doi: 10.2196/48244 (PMC10770782; doi:10.2196/48244)
Supplement: Multimedia Appendix 8 [file jmir_v25i1e48244_app8.docx]

**Multimedia Appendix 8.** Performance metrics of most models using the 24-hour time window.

**Table S1.** Statistical Comparisons Results of Performance Metrics among Different Features Sets with 95% Confidence Interval Values by Logistic Regression using 24-hour Time Step Window from MIMIC^a^-IV.

| **Performance Metrics** | **Feature Set** | **95% CI**^b^ | | ***P* value** |
| --- | --- | --- | --- | --- |
|  |  | **Lower limit** | **Upper limit** |  |
| **Precision** | **Statistical vs Similarity** | 0.02 | 0.18 | .01 |
|  | **All vs Statistical** | 0.01 | 0.16 | .03 |
|  | **All vs Similarity** | -0.09 | 0.07 | .90 |
| **F1-score** | **Statistical vs Similarity** | 0.14 | 0.34 | <.001 |
|  | **All vs Statistical** | 0.12 | 0.33 | <.001 |
|  | **All vs Similarity** | -0.12 | 0.09 | .90 |
| **AUPRC**^c^ | **Statistical vs Similarity** | 0.02 | 0.54 | .03 |
|  | **All vs Statistical** | -0.07 | 0.44 | .19 |
|  | **All vs Similarity** | -0.35 | 0.16 | .63 |

^a^MIMIC: medical information mart for intensive care

^b^CI: confidence interval

^c^AUPRC: area under the precision-recall curve

**Table S2.** Statistical Comparisons Results of Performance Metrics among Different Features Sets with 95% Confidence Interval Values by Decision Tree using 24-hour Time Step Window from MIMIC^a^-IV.

| **Performance Metrics** | **Feature Set** | **95% CI**^b^ | | ***P* value** |
| --- | --- | --- | --- | --- |
|  |  | **Lower limit** | **Upper limit** |  |
| **Precision** | **Statistical vs Similarity** | -0.45 | -0.13 | <.001 |
|  | **All vs Statistical** | -0.45 | -0.13 | <.001 |
|  | **All vs Similarity** | -0.16 | 0.16 | .90 |
| **F1-score** | **Statistical vs Similarity** | -0.10 | -0.02 | <.001 |
|  | **All vs Statistical** | -0.10 | -0.02 | <.001 |
|  | **All vs Similarity** | -0.04 | 0.04 | .90 |

^a^MIMIC: medical information mart for intensive care

^b^CI: confidence interval

**Table S3.** Statistical Comparisons Results of Performance Metrics among Different Features Sets with 95% Confidence Interval Values by Gaussian naïve Bayes using 24-hour Time Step Window from MIMIC^a^-IV.

| **Performance Metrics** | **Feature Set** | **95% CI**^b^ | | ***P* value** |
| --- | --- | --- | --- | --- |
|  |  | **Lower limit** | **Upper limit** |  |
| **Precision** | **Statistical vs Similarity** | 0.02 | 0.06 | <.001 |
|  | **All vs Statistical** | 0.00 | 0.04 | .02 |
|  | **All vs Similarity** | -0.04 | 0.00 | .04 |
| **Sensitivity** | **Statistical vs Similarity** | 0.01 | 0.17 | .02 |
|  | **All vs Statistical** | -0.01 | 0.15 | .08 |
|  | **All vs Similarity** | -0.10 | 0.06 | .77 |
| **Specificity** | **Statistical vs Similarity** | 0.01 | 0.17 | .02 |
|  | **All vs Statistical** | -0.01 | 0.15 | .08 |
|  | **All vs Similarity** | -0.10 | 0.06 | .77 |
| **F1-score** | **Statistical vs Similarity** | 0.09 | 0.14 | <.001 |
|  | **All vs Statistical** | 0.04 | 0.10 | <.001 |
|  | **All vs Similarity** | -0.07 | -0.01 | <.001 |
| **AUROC**^c^ | **Statistical vs Similarity** | 0.01 | 0.17 | .02 |
|  | **All vs Statistical** | -0.01 | 0.15 | .08 |
|  | **All vs Similarity** | -0.10 | 0.06 | .77 |

^a^MIMIC: medical information mart for intensive care

^b^CI: confidence interval

^c^AUROC: area under the receiver operating characteristic curve

**Table S4.** Statistical Comparisons Results of Performance Metrics among Different Features Sets with 95% Confidence Interval Values by Random Forest using 24-hour Time Step Window from MIMIC^a^-IV.

| **Performance Metrics** | **Feature Set** | **95% CI**^b^ | | ***P* value** |
| --- | --- | --- | --- | --- |
|  |  | **Lower limit** | **Upper limit** |  |
| **Sensitivity** | **Statistical vs Similarity** | 0.00 | 0.03 | .06 |
|  | **All vs Statistical** | 0.07 | 0.10 | <.001 |
|  | **All vs Similarity** | 0.06 | 0.09 | <.001 |
| **Specificity** | **Statistical vs Similarity** | 0.00 | 0.03 | .06 |
|  | **All vs Statistical** | 0.07 | 0.10 | <.001 |
|  | **All vs Similarity** | 0.06 | 0.09 | <.001 |
| **F1-score** | **Statistical vs Similarity** | 0.00 | 0.07 | .02 |
|  | **All vs Statistical** | 0.11 | 0.18 | <.001 |
|  | **All vs Similarity** | 0.07 | 0.14 | <.001 |
| **AUROC**^c^ | **Statistical vs Similarity** | 0.00 | 0.03 | .06 |
|  | **All vs Statistical** | 0.07 | 0.10 | <.001 |
|  | **All vs Similarity** | 0.06 | 0.09 | <.001 |
| **AUPRC**^d^ | **Statistical vs Similarity** | 0.13 | 0.46 | <.001 |
|  | **All vs Statistical** | 0.11 | 0.45 | <.001 |
|  | **All vs Similarity** | -0.18 | 0.15 | .90 |

^a^MIMIC: medical information mart for intensive care

^b^CI: confidence interval

^c^AUROC: area under the receiver operating characteristic curve

^d^AUPRC: area under the precision-recall curve

**Table S5.** Statistical Comparisons Results of Performance Metrics among Different Features Sets with 95% Confidence Interval Values by extreme Gradient Boosting Ensemble of Decision Trees using 24-hour Time Step Window from MIMIC^a^-IV.

| **Performance Metrics** | **Feature Set** | **95% CI**^b^ | | ***P* value** |
| --- | --- | --- | --- | --- |
|  |  | **Lower limit** | **Upper limit** |  |
| **Precision** | **Statistical vs Similarity** | 0.03 | 0.05 | <.001 |
|  | **All vs Statistical** | 0.03 | 0.06 | <.001 |
|  | **All vs Similarity** | 0.00 | 0.02 | .31 |
| **Sensitivity** | **Statistical vs Similarity** | 0.07 | 0.21 | <.001 |
|  | **All vs Statistical** | 0.09 | 0.23 | <.001 |
|  | **All vs Similarity** | -0.05 | 0.08 | .83 |
| **Specificity** | **Statistical vs Similarity** | 0.07 | 0.21 | <.001 |
|  | **All vs Statistical** | 0.09 | 0.23 | <.001 |
|  | **All vs Similarity** | -0.05 | 0.08 | .83 |
| **F1-score** | **Statistical vs Similarity** | 0.13 | 0.17 | <.001 |
|  | **All vs Statistical** | 0.15 | 0.19 | <.001 |
|  | **All vs Similarity** | -0.01 | 0.04 | .20 |
| **AUROC**^c^ | **Statistical vs Similarity** | 0.07 | 0.21 | <.001 |
|  | **All vs Statistical** | 0.09 | 0.23 | <.001 |
|  | **All vs Similarity** | -0.05 | 0.08 | .83 |

^a^MIMIC: medical information mart for intensive care

^b^CI: confidence interval

^c^AUROC: area under the receiver operating characteristic curve

**Table S6.** Statistical Comparisons Results of Performance Metrics among Different Features Sets with 95% Confidence Interval Values by Gradient Boosting Ensemble of Decision Trees using 24-hour Time Step Window from MIMIC^a^-IV.

| **Performance Metrics** | **Feature Set** | **95% CI**^b^ | | ***P* value** |
| --- | --- | --- | --- | --- |
|  |  | **Lower limit** | **Upper limit** |  |
| **Precision** | **Statistical vs Similarity** | 0.01 | 0.03 | <.001 |
|  | **All vs Statistical** | 0.01 | 0.03 | <.001 |
|  | **All vs Similarity** | -0.01 | 0.01 | .64 |
| **Sensitivity** | **Statistical vs Similarity** | 0.05 | 0.17 | <.001 |
|  | **All vs Statistical** | 0.07 | 0.18 | <.001 |
|  | **All vs Similarity** | -0.04 | 0.07 | .79 |
| **Specificity** | **Statistical vs Similarity** | 0.05 | 0.17 | <.001 |
|  | **All vs Statistical** | 0.07 | 0.18 | <.001 |
|  | **All vs Similarity** | -0.04 | 0.07 | .79 |
| **F1-score** | **Statistical vs Similarity** | 0.11 | 0.15 | <.001 |
|  | **All vs Statistical** | 0.12 | 0.16 | <.001 |
|  | **All vs Similarity** | -0.01 | 0.03 | .27 |
| **AUROC**^c^ | **Statistical vs Similarity** | 0.05 | 0.17 | <.001 |
|  | **All vs Statistical** | 0.07 | 0.18 | <.001 |
|  | **All vs Similarity** | -0.04 | 0.07 | .79 |

^a^MIMIC: medical information mart for intensive care

^b^CI: confidence interval

^c^AUROC: area under the receiver operating characteristic curve

**Table S7.** Statistical Comparisons Results of Performance Metrics among Different Features Sets with 95% Confidence Interval Values by a Proposed Method using 24-hour Time Step Window from MIMIC^a^-IV.

| **Performance Metrics** | **Feature Set** | **95% CI**^b^ | | ***P* value** |
| --- | --- | --- | --- | --- |
|  |  | **Lower limit** | **Upper limit** |  |
| **Precision** | **Statistical vs Similarity** | -0.05 | 0.11 | .57 |
|  | **All vs Statistical** | 0.08 | 0.23 | <.001 |
|  | **All vs Similarity** | 0.05 | 0.20 | <.001 |
| **Sensitivity** | **Statistical vs Similarity** | 0.07 | 0.22 | <.001 |
|  | **All vs Statistical** | 0.15 | 0.30 | <.001 |
|  | **All vs Similarity** | 0.01 | 0.16 | .03 |
| **Specificity** | **Statistical vs Similarity** | 0.07 | 0.22 | <.001 |
|  | **All vs Statistical** | 0.15 | 0.29 | <.001 |
|  | **All vs Similarity** | 0.00 | 0.15 | .04 |
| **F1-score** | **Statistical vs Similarity** | 0.06 | 0.24 | <.001 |
|  | **All vs Statistical** | 0.25 | 0.43 | <.001 |
|  | **All vs Similarity** | 0.10 | 0.28 | <.001 |
| **AUROC**^c^ | **Statistical vs Similarity** | 0.09 | 0.20 | <.001 |
|  | **All vs Statistical** | 0.12 | 0.24 | <.001 |
|  | **All vs Similarity** | -0.02 | 0.10 | .26 |

^a^MIMIC: medical information mart for intensive care

^b^CI: confidence interval

^c^AUROC: area under the receiver operating characteristic curve
