# Supplementary material for: Explainable Artificial Intelligence Warning Model Using an Ensemble Approach for In-Hospital Cardiac Arrest Prediction: Retrospective Cohort Study
Source: J Med Internet Res. 2023 Dec 22;25:e48244. doi: 10.2196/48244 (PMC10770782; doi:10.2196/48244)
Supplement: Multimedia Appendix 9 [file jmir_v25i1e48244_app9.docx]

**Multimedia Appendix 9.** Performance metrics of most models using the 12-hour time window.

**Table S1.** Statistical Comparisons Results of Performance Metrics among Different Features Sets with 95% Confidence Interval Values by Logistic Regression using 12-hour Time Step Window from MIMIC^a^-IV.

| **Performance Metrics** | **Feature Set** | **95% CI**^b^ | | ***P* value** |
| --- | --- | --- | --- | --- |
|  |  | **Lower limit** | **Upper limit** |  |
| **F1-score** | **Statistical vs Similarity** | 0.08 | 0.16 | <.001 |
|  | **All vs Statistical** | 0.11 | 0.19 | <.001 |
|  | **All vs Similarity** | -0.01 | 0.07 | .15 |

^a^MIMIC: medical information mart for intensive care

^b^CI: confidence interval

**Table S2.** Statistical Comparisons Results of Performance Metrics among Different Features Sets with 95% Confidence Interval Values by K-Nearest Neighbors using 12-hour Time Step Window from MIMIC^a^-IV.

| **Performance Metrics** | **Feature Set** | **95% CI**^b^ | | ***P* value** |
| --- | --- | --- | --- | --- |
|  |  | **Lower limit** | **Upper limit** |  |
| **Precision** | **Statistical vs Similarity** | 0.11 | 0.56 | <.001 |
|  | **All vs Statistical** | 0.04 | 0.49 | .02 |
|  | **All vs Similarity** | -0.30 | 0.15 | .71 |
| **Specificity** | **Statistical vs Similarity** | 0.01 | 0.08 | .02 |
|  | **All vs Statistical** | 0.01 | 0.09 | .01 |
|  | **All vs Similarity** | -0.03 | 0.04 | .90 |
| **F1-score** | **Statistical vs Similarity** | 0.02 | 0.14 | .01 |
|  | **All vs Statistical** | 0.02 | 0.14 | .01 |
|  | **All vs Similarity** | -0.06 | 0.06 | .90 |
| **AUROC**^c^ | **Statistical vs Similarity** | 0.01 | 0.08 | .02 |
|  | **All vs Statistical** | 0.01 | 0.09 | .01 |
|  | **All vs Similarity** | -0.03 | 0.04 | .90 |
| **AUPRC**^d^ | **Statistical vs Similarity** | 0.01 | 0.08 | .02 |
|  | **All vs Statistical** | 0.01 | 0.09 | .01 |
|  | **All vs Similarity** | -0.03 | 0.04 | .90 |

^a^MIMIC: medical information mart for intensive care

^b^CI: confidence interval

^c^AUROC: area under the receiver operating characteristic curve

^d^AUPRC: area under the precision-recall curve

**Table S3.** Statistical Comparisons Results of Performance Metrics among Different Features Sets with 95% Confidence Interval Values by Decision Tree using 12-hour Time Step Window from MIMIC^a^-IV.

| **Performance Metrics** | **Feature Set** | **95% CI**^b^ | | ***P* value** |
| --- | --- | --- | --- | --- |
|  |  | **Lower limit** | **Upper limit** |  |
| **Precision** | **Statistical vs Similarity** | -0.51 | -0.50 | <.001 |
|  | **All vs Statistical** | -0.51 | -0.50 | <.001 |
|  | **All vs Similarity** | 0.00 | 0.00 | .90 |
| **Specificity** | **Statistical vs Similarity** | -0.08 | -0.06 | <.001 |
|  | **All vs Statistical** | -0.08 | -0.06 | <.001 |
|  | **All vs Similarity** | -0.01 | 0.01 | .90 |
| **F1-score** | **Statistical vs Similarity** | -0.15 | -0.12 | <.001 |
|  | **All vs Statistical** | -0.15 | -0.12 | <.001 |
|  | **All vs Similarity** | -0.01 | 0.01 | .90 |
| **AUROC**^c^ | **Statistical vs Similarity** | -0.08 | -0.06 | <.001 |
|  | **All vs Statistical** | -0.08 | -0.06 | <.001 |
|  | **All vs Similarity** | -0.01 | 0.01 | .90 |
| **AUPRC**^d^ | **Statistical vs Similarity** | -0.08 | -0.06 | <.001 |
|  | **All vs Statistical** | -0.08 | -0.06 | <.001 |
|  | **All vs Similarity** | -0.01 | 0.01 | .90 |

^a^MIMIC: medical information mart for intensive care

^b^CI: confidence interval

^c^AUROC: area under the receiver operating characteristic curve

^d^AUPRC: area under the precision-recall curve

**Table S4.** Statistical Comparisons Results of Performance Metrics among Different Features Sets with 95% Confidence Interval Values by Support Vector Machine using 12-hour Time Step Window from MIMIC^a^-IV.

| **Performance Metrics** | **Feature Set** | **95% CI**^b^ | | ***P* value** |
| --- | --- | --- | --- | --- |
|  |  | **Lower limit** | **Upper limit** |  |
| **Precision** | **Statistical vs Similarity** | -0.04 | 0.12 | .41 |
|  | **All vs Statistical** | 0.04 | 0.20 | <.001 |
|  | **All vs Similarity** | 0.00 | 0.16 | .05 |
| **F1-score** | **Statistical vs Similarity** | 0.01 | 0.21 | .04 |
|  | **All vs Statistical** | 0.10 | 0.31 | <.001 |
|  | **All vs Similarity** | -0.01 | 0.20 | .08 |
| **AUPRC**^c^ | **Statistical vs Similarity** | -0.14 | 0.28 | .67 |
|  | **All vs Statistical** | 0.07 | 0.49 | .01 |
|  | **All vs Similarity** | 0.00 | 0.42 | .05 |

^a^MIMIC: medical information mart for intensive care

^b^CI: confidence interval

^c^AUPRC: area under the precision-recall curve

**Table S5.** Statistical Comparisons Results of Performance Metrics among Different Features Sets with 95% Confidence Interval Values by Gaussian naïve Bayes using 12-hour Time Step Window from MIMIC^a^-IV.

| **Performance Metrics** | **Feature Set** | **95% CI**^b^ | | ***P* value** |
| --- | --- | --- | --- | --- |
|  |  | **Lower limit** | **Upper limit** |  |
| **Precision** | **Statistical vs Similarity** | 0.00 | 0.04 | .07 |
|  | **All vs Statistical** | 0.00 | 0.04 | .05 |
|  | **All vs Similarity** | -0.02 | 0.02 | .90 |
| **F1-score** | **Statistical vs Similarity** | 0.05 | 0.12 | <.001 |
|  | **All vs Statistical** | 0.04 | 0.11 | <.001 |
|  | **All vs Similarity** | -0.05 | 0.02 | .62 |

^a^MIMIC: medical information mart for intensive care

^b^CI: confidence interval

**Table S6.** Statistical Comparisons Results of Performance Metrics among Different Features Sets with 95% Confidence Interval Values by Random Forest using 12-hour Time Step Window from MIMIC^a^-IV.

| **Performance Metrics** | **Feature Set** | **95% CI**^b^ | | ***P* value** |
| --- | --- | --- | --- | --- |
|  |  | **Lower limit** | **Upper limit** |  |
| **Precision** | **Statistical vs Similarity** | 0.34 | 0.54 | <.001 |
|  | **All vs Statistical** | 0.34 | 0.55 | <.001 |
|  | **All vs Similarity** | -0.10 | 0.11 | .90 |
| **F1-score** | **Statistical vs Similarity** | 0.02 | 0.07 | <.001 |
|  | **All vs Statistical** | 0.03 | 0.07 | <.001 |
|  | **All vs Similarity** | -0.02 | 0.03 | .81 |
| **AUPRC**^c^ | **Statistical vs Similarity** | 0.04 | 0.32 | .01 |
|  | **All vs Statistical** | 0.04 | 0.32 | .01 |
|  | **All vs Similarity** | -0.14 | 0.14 | .90 |

^a^MIMIC: medical information mart for intensive care

^b^CI: confidence interval

^c^AUPRC: area under the precision-recall curve

**Table S7.** Statistical Comparisons Results of Performance Metrics among Different Features Sets with 95% Confidence Interval Values by extreme Gradient Boosting Ensemble of Decision Trees using 12-hour Time Step Window from MIMIC^a^-IV.

| **Performance Metrics** | **Feature Set** | **95% CI**^b^ | | ***P* value** |
| --- | --- | --- | --- | --- |
|  |  | **Lower limit** | **Upper limit** |  |
| **Precision** | **Statistical vs Similarity** | 0.00 | 0.02 | .09 |
|  | **All vs Statistical** | 0.00 | 0.03 | .01 |
|  | **All vs Similarity** | -0.01 | 0.02 | .61 |
| **Specificity** | **Statistical vs Similarity** | 0.00 | 0.13 | .04 |
|  | **All vs Statistical** | 0.02 | 0.15 | .01 |
|  | **All vs Similarity** | -0.04 | 0.08 | .66 |
| **F1-score** | **Statistical vs Similarity** | 0.04 | 0.09 | <.001 |
|  | **All vs Statistical** | 0.08 | 0.12 | <.001 |
|  | **All vs Similarity** | 0.02 | 0.06 | <.001 |
| **AUROC**^c^ | **Statistical vs Similarity** | 0.00 | 0.13 | .04 |
|  | **All vs Statistical** | 0.02 | 0.15 | .01 |
|  | **All vs Similarity** | -0.04 | 0.08 | .66 |
| **AUPRC**^d^ | **Statistical vs Similarity** | 0.00 | 0.13 | .04 |
|  | **All vs Statistical** | 0.02 | 0.15 | .01 |
|  | **All vs Similarity** | -0.04 | 0.08 | .66 |

^a^MIMIC: medical information mart for intensive care

^b^CI: confidence interval

^c^AUROC: area under the receiver operating characteristic curve

^d^AUPRC: area under the precision-recall curve

**Table S8.** Statistical Comparisons Results of Performance Metrics among Different Features Sets with 95% Confidence Interval Values by Gradient Boosting Ensemble of Decision Trees using 12-hour Time Step Window from MIMIC^a^-IV.

| **Performance Metrics** | **Feature Set** | **95% CI**^b^ | | ***P* value** |
| --- | --- | --- | --- | --- |
|  |  | **Lower limit** | **Upper limit** |  |
| **Specificity** | **Statistical vs Similarity** | -0.02 | 0.07 | .37 |
|  | **All vs Statistical** | 0.03 | 0.12 | <.001 |
|  | **All vs Similarity** | 0.00 | 0.09 | .05 |
| **F1-score** | **Statistical vs Similarity** | 0.02 | 0.06 | <.001 |
|  | **All vs Statistical** | 0.07 | 0.11 | <.001 |
|  | **All vs Similarity** | 0.03 | 0.08 | <.001 |
| **AUROC**^c^ | **Statistical vs Similarity** | -0.02 | 0.07 | .37 |
|  | **All vs Statistical** | 0.03 | 0.12 | <.001 |
|  | **All vs Similarity** | 0.00 | 0.09 | .05 |
| **AUPRC**^d^ | **Statistical vs Similarity** | -0.02 | 0.07 | .37 |
|  | **All vs Statistical** | 0.03 | 0.12 | <.001 |
|  | **All vs Similarity** | 0.00 | 0.09 | .05 |

^a^MIMIC: medical information mart for intensive care

^b^CI: confidence interval

^c^AUROC: area under the receiver operating characteristic curve

^d^AUPRC: area under the precision-recall curve

**Table S9.** Statistical Comparisons Results of Performance Metrics among Different Features Sets with 95% Confidence Interval Values by a Proposed Method using 12-hour Time Step Window from MIMIC^a^-IV.

| **Performance Metrics** | **Feature Set** | **95% CI**^b^ | | ***P* value** |
| --- | --- | --- | --- | --- |
|  |  | **Lower limit** | **Upper limit** |  |
| **Precision** | **Statistical vs Similarity** | 0.00 | 0.01 | .59 |
|  | **All vs Statistical** | 0.00 | 0.02 | .02 |
|  | **All vs Similarity** | 0.00 | 0.01 | .18 |
| **Specificity** | **Statistical vs Similarity** | -0.01 | 0.08 | .18 |
|  | **All vs Statistical** | 0.03 | 0.11 | <.001 |
|  | **All vs Similarity** | -0.01 | 0.08 | .11 |
| **F1-score** | **Statistical vs Similarity** | 0.04 | 0.08 | <.001 |
|  | **All vs Statistical** | 0.08 | 0.12 | <.001 |
|  | **All vs Similarity** | 0.03 | 0.07 | <.001 |
| **AUROC**^c^ | **Statistical vs Similarity** | -0.01 | 0.08 | .18 |
|  | **All vs Statistical** | 0.03 | 0.11 | <.001 |
|  | **All vs Similarity** | -0.01 | 0.08 | .11 |
| **AUPRC**^d^ | **Statistical vs Similarity** | -0.01 | 0.08 | .18 |
|  | **All vs Statistical** | 0.03 | 0.11 | <.001 |
|  | **All vs Similarity** | -0.01 | 0.08 | .11 |

^a^MIMIC: medical information mart for intensive care

^b^CI: confidence interval

^c^AUROC: area under the receiver operating characteristic curve

^d^AUPRC: area under the precision-recall curve
