# Supplementary material for: Explainable Artificial Intelligence Warning Model Using an Ensemble Approach for In-Hospital Cardiac Arrest Prediction: Retrospective Cohort Study
Source: J Med Internet Res. 2023 Dec 22;25:e48244. doi: 10.2196/48244 (PMC10770782; doi:10.2196/48244)
Supplement: Multimedia Appendix 10 [file jmir_v25i1e48244_app10.docx]

**Multimedia Appendix 10.** Comparison results of performance metrics between the proposed method and the baseline models using the eICU Collaborative Research Database.

|  | **Precision**  (95% CI^j^) | **Sensitivity**  (95% CI) | **Specificity**  (95% CI) | **F1-score**  (95% CI) | **AUROC^j^**  (95% CI) | **AUPRC^k^**  (95% CI) | **Brier Score**  (95% CI) |
| --- | --- | --- | --- | --- | --- | --- | --- |
| **LR**^a^ | 0.50  (0.50 - 0.51) | 0.52  (0.50 - 0.55) | 0.52  (0.50 - 0.55) | 0.49  (0.48 - 0.49) | 0.52  (0.46 - 0.53) | 0.03  (0.02 - 0.05) | 0.12  (0.11 - 0.12) |
| **KNN**^b^ | 0.57  (0.523 - 0.61) | 0.52  (0.51 - 0.53) | 0.519  (0.51 - 0.53) | 0.53  (0.51 - 0.55) | 0.52  (0.54 - 0.59) | 0.05  (0.04 - 0.08) | 0.03  (0.02 - 0.03) |
| **DT**^c^ | 0.01  (0.01 - 0.01) | 0.50  (0.50 - 0.50) | 0.50  (0.50 - 0.50) | 0.02  (0.02 - 0.02) | 0.50  (0.56 - 0.60) | 0.16  (0.13 - 0.20) | 0.57  (0.57 - 0.57) |
| **SVM**^d^ | 0.51  (0.50 - 0.52) | 0.52  (0.51 - 0.54) | 0.52  (0.51 - 0.54) | 0.51  (0.50 - 0.53) | 0.52  (0.46 - 0.53) | 0.04  (0.02 - 0.05) | 0.02  (0.02 - 0.02) |
| **GB**^e^ | 0.51  (0.51 - 0.51) | 0.61  (0.58 - 0.64) | 0.61  (0.58 - 0.64) | 0.42  (0.42 - 0.43) | 0.61  (0.61 - 0.67) | 0.25  (0.22 - 0.29) | 0.36  (0.35 - 0.36) |
| **MLP**^f^ | 0.57  (0.55 - 0.60) | 0.55  (0.53 - 0.56) | 0.55  (0.53 - 0.56) | 0.56  (0.54 - 0.58) | 0.55  (0.58 - 0.65) | 0.07  (0.05 - 0.10) | 0.03  (0.02 - 0.03) |
| **RF**^g^ | 0.01  (0.01 - 0.01) | 0.50  (0.50 - 0.50) | 0.50  (0.50 - 0.50) | 0.02  (0.02 - 0.02) | 0.50  (0.61 - 0.67) | 0.11  (0.08 - 0.14) | 0.48  (0.47 - 0.48) |
| **XGB**^h^ | 0.51  (0.51 - 0.51) | 0.63  (0.60 - 0.66) | 0.63  (0.60 - 0.66) | 0.41  (0.41 - 0.42) | 0.63  (0.68 - 0.76) | 0.44  (0.38 - 0.49) | 0.21  (0.21 - 0.21) |
| **LGB**^i^ | 0.51  (0.51 - 0.51) | 0.600  (0.58 - 0.63) | 0.60  (0.58 - 0.63) | 0.37  (0.37 - 0.38) | 0.60  (0.66 - 0.74) | 0.41  (0.36 - 0.47) | 0.24  (0.24 - 0.24) |
| **Proposed method** | **0.59**  **(0.58 - 0.61)** | **0.74**  **(0.71 - 0.76)** | **0.92**  **(0.91 - 0.94)** | **0.63**  **(0.61 - 0.65)** | **0.74**  **(0.70 - 0.77)** | **0.44**  **(0.38 - 050)** | **0.11**  **(0.11 - 0.12)** |

^a^LR: logistic regression

^b^KNN: k-nearest neighbors

^c^DT: decision tree

^d^SVM: support vector machine

^e^GB: Gaussian naïve Bayes

^f^MLP: multilayer perceptron

^g^RF: random forest

^h^XGB: extreme gradient boosting ensemble of decision trees

^i^LGB: gradient boosting ensemble of decision trees

^j^AUROC: area under the receiver operating characteristic curve

^k^AUPRC: area under the precision-recall curve

^l^CI: confidence interval
