# Supplementary material for: Explainable Artificial Intelligence Warning Model Using an Ensemble Approach for In-Hospital Cardiac Arrest Prediction: Retrospective Cohort Study
Source: J Med Internet Res. 2023 Dec 22;25:e48244. doi: 10.2196/48244 (PMC10770782; doi:10.2196/48244)
Supplement: Multimedia Appendix 11 [file jmir_v25i1e48244_app11.docx]

**Multimedia Appendix 11.** Comparison results of performance metrics between the proposed method and methods in recent studies.

| **Author** | **Precision** | **Sensitivity** | **Specificity** | **F1-score** | **AUROC^a^** | **AUPRC^b^** |
| --- | --- | --- | --- | --- | --- | --- |
| **Churpek et al. [14]** | - | - | - | - | 0.8 | - |
| **Kwon**  **et al. [18]** | - | 0.76 | 0.77 | - | 0.85 | 0.04 |
| **Layeghian Javan**  **et al. [16]** | 0.19 | 0.77 | 0.76 | 0.31 | 0.82 | - |
| **Proposed method** | **0.68** | **0.90** | **0.90** | **0.72** | **0.86** | **0.58** |

^a^AUROC: area under the receiver operating characteristic curve

^b^AUPRC: area under the precision-recall curve
